# Supplementary material for: Tousled-like kinase 2 targets ASF1 histone chaperones through client mimicry
Source: Nat Commun. 2022 Feb 8;13:749. doi: 10.1038/s41467-022-28427-0 (PMC8826447; doi:10.1038/s41467-022-28427-0)
Supplement: Supplementary file 1 — Supplementary Information [file 41467_2022_28427_MOESM1_ESM.pdf]

Supplementary information

Tousled-like kinase 2 targets ASF1 histone chaperones through client mimicry

Bertrand Simon[1], Hua Jane Lou[1], Clotilde Huet-Calderwood[1], Guangda Shi[1], Titus J. Boggon[1,2], Benjamin E. Turk[1,†] and David A. Calderwood[1,3,†]

[1] Department of Pharmacology, Yale School of Medicine, New Haven, Connecticut, United States of America.
[2] Department of Molecular Biophysics and Biochemistry, Yale University, New Haven, Connecticut, United States of America.
[3] Department of Cell Biology, Yale School of Medicine, New Haven, Connecticut, United States of America.
[4] Corresponding authors: ben.turk@yale.edu or david.calderwood@yale.edu

Supplementary Tables 1 & 2
Supplementary Figures 1-5

Supplementary Table 1. Quantified PSPL data. Spot intensities were normalized by position to an average value of 1. Data show the mean of two separate experiments.

		Position									
		-5	-4	-3	-2	-1	0	+1	+2	+3	+4
Amino acid residue	P	0.44	0.32	0.50	0.27	0.00		0.00	0.64	1.03	1.55
	G	0.53	0.52	0.52	0.51	0.21		0.03	0.61	1.48	0.78
	A	0.66	1.07	0.50	0.58	0.68		0.20	0.52	1.22	0.66
	C	1.51	1.09	1.29	0.45	0.63		1.02	1.06	0.95	1.30
	S	0.75	0.95	1.53	0.66	0.64	0.87	1.60	2.04	2.05	0.82
	T	0.84	1.00	1.13	1.31	0.67	1.13	1.50	1.53	2.13	0.78
	V	0.98	0.58	0.34	0.20	0.56		1.40	0.37	0.42	0.78
	I	0.74	1.05	0.58	0.28	0.73		1.29	0.71	0.61	1.26
	L	1.00	1.42	0.53	0.21	0.53		1.37	0.94	0.67	1.08
	M	0.98	0.85	0.83	0.34	0.51		2.86	1.70	0.86	1.08
	F	2.00	1.55	0.62	0.26	0.30		0.28	0.60	1.20	2.20
	Y	1.49	1.62	1.53	0.42	0.49		0.16	0.86	1.28	1.22
	W	2.21	1.63	0.77	0.37	0.60		0.28	1.61	0.99	2.43
	H	0.89	1.30	0.77	0.70	0.98		0.70	1.81	0.87	0.90
	K	0.25	0.19	0.23	0.10	0.21		0.17	0.16	0.04	0.46
	R	0.66	0.31	0.35	0.29	0.35		1.29	0.50	0.15	0.42
	Q	0.55	0.63	0.66	0.82	0.91		2.75	1.81	0.92	0.33
	N	0.98	1.25	1.68	0.99	2.08		2.98	0.69	0.76	0.79
	D	1.47	1.58	3.58	4.54	3.85		0.01	1.36	1.02	0.64
	E	1.08	1.10	2.07	6.70	5.16		0.21	0.47	1.36	0.53
	pT	0.58	1.35	1.62	1.03	0.86		0.35	0.99	1.28	0.52
	pY	0.45	1.29	1.19	2.27	3.03		0.33	1.61	1.36	0.91

Supplementary Table 2. Primers used in this study.

Name	Sequence
Forward PCR primer for full-length TLK2	TACAGAGCTCAAGCTTATGGAAGAATTGCATAGCCTG
Reverse PCR primer for full-length TLK2	ATTACCGCGGTACCTCATTAATTAGAAGAACTGTTATTGGACGCC
Forward PCR primer for TLK2 123-	TACAGAGCTCAAGCTTCGAGTAGAACAGCCCCTCTATG
Forward PCR primer for TLK2 214-	TACAGAGCTCAAGCTTATAGAAAAAATATCTGCACTAGAAAACAGTAAGAATTC
Forward PCR primer for TLK2 396-	TACAGAGCTCAAGCTTAGGTTAACGTTAGCAGAATACCATG
Forward PCR primer for TLK2 ΔN178	CTCAGGATCCAGCTCAGCAAAACAGTCCCTC
Reverse PCR primer for TLK2 C-terminus	GACTGCGGCCGCTCAATTAGAAGAACTGTTATTGGACGC
TLK2 Stop 451 QuikChange primer 1	CATAATGAAGATAATTCATGATAAAAAGATCATCCAACGC
TLK2 Stop 451 QuikChange primer 2	GCGTTGGATGATCTTTTTATCATGAATTATCTTCATTATG
TLK2 Stop 396 QuikChange primer 1	CCCACAGTTTACTTTGATAAACGTTAGCAGAATACC
TLK2 Stop 396 QuikChange primer 2	GGTATTCTGCTAACGTTTATCAAAGTAAACTGTGGG
TLK2 Stop 296 QuikChange primer 1	CAAGACCGCTTGAGATAATGACACTTTACTACTGTC
TLK2 Stop 296 QuikChange primer 2	GACAGTAGTAAAGTGTCATTATCTCAAGCGGTCTTG
TLK2 Stop 214 QuikChange primer 1	CAGTCCGACCTCACATGATAAAAAATATCTGCACTAG
TLK2 Stop 214 QuikChange primer 2	CTAGTGCAGATATTTTTTATCATGTGAGGTCGGACTG
TLK2 Stop 123 QuikChange primer 1	CAATCCCTTACCGCGATGATAAGAACAGCCCCTC
TLK2 Stop 123 QuikChange primer 2	CCATAGAGGGGCTGTTCTTATCATCGCGGTAAGG
TLK2 D592A QuikChange primer 1	AATATTACCTGGTTTGAGGGCATAGTGTATGATGGGAGG
TLK2 D592A QuikChange primer 2	CCTCCCATCATACACTATGCCCTCAAACCAGGTAATATT
TLK2 L8R QuikChange primer 1	GAAGAATTGCATAGCCGGGACCCACGAC
TLK2 L8R QuikChange primer 2	GTCGTGGGTCCCGGCTATGCAATTCTTC
TLK2 R11A QuikChange primer 1	CATAGCCTGGACCCAGCACGGCAGGAATTATTG
TLK2 R11A QuikChange primer 2	CAATAATTCCTGCCGTGCTGGGTCCAGGCTATG
TLK2 R12A QuikChange primer 1	CTGGACCCACGAGCGCAGGAATTATTGGAGG
TLK2 R12A QuikChange primer 2	CCTCCAATAATTCCTGCGCTCGTGGGTCCAG
TLK2 L16A/R19A/F20A QuikChange primer 1	GACGGCAGGAATTAGCGGAGGCCGCGGCTACTGGAGTAGGTGTTAG
TLK2 L16A/R19A/F20A QuikChange primer 2	CTAACACCTACTCCAGTAGCCGCGGCCTCCGCTAATTCCTGCCGTC
Forward PCR primer for ASF1a	CTCTGGATCCATGGCAAAGGTTCAGGTGAAC
Forward PCR primer for ASF1a-CT (155-204)	CTACGGATCCGATAACACAGAAAAACTGGAAGATG
Reverse PCR primer for ASF1a-NT (1-155)	GAGAGCGGCCGCTCAATCTTCCCAATTAATGTGGAATCTTG
Reverse PCR primer for ASF1a full-length	CAGAGCGGCCGCTCACATGCAGTCCATGTGG
Forward PCR primer for ASF1b	GTCTGGATCCATGGCCAAGGTGTCGGTG
Forward PCR primer for ASF1b-CT (155-204)	GTGAGGATCCAACAACATGGACAGGCTGG
Reverse PCR primer for ASF1b-NT (1-155)	GAGAGCGGCCGCTCAGTTGTCCCAGTTGATATGGAAG
Reverse PCR primer for ASF1b full-length	CTGAGCGGCCGCTTAGATGCAGTCCATGGAGTTC

ASF1a V94R QuikChange primer 1	GCAGTAGGCGTAACTCGTGTGCTAATTACTTGTAC
ASF1a V94R QuikChange primer 2	GTACAAGTAATTAGCACACGAGTTACGCCTACTGC
ASF1a D37A QuikChange primer 1	GACCTGTCTGAAGCCTTGGAATGGAAAATTATC
ASF1a D37A QuikChange primer 2	GATAATTTTCCATTCCAAGGCTTCAGACAGGTC
ASF1a E49A QuikChange primer 1	CTATGTGGGCTCTGCAGCAAGTGAAGAATAC
ASF1a E49A QuikChange primer 2	GTATTCTTCACTTGCTGCAGAGCCCACATAG
ASF1a D58A QuikChange primer 1	CGATCAAGTTTTAGCCTCTGTTTTAGTGGGTCC
ASF1a D58A QuikChange primer 2	GGACCCACTAAAACAGAGGCTAAAACTTGATCG
ASF1a D88A QuikChange primer 1	GGACTCATTCCAGATGCAGCTGCAGTAGGC
ASF1a D88A QuikChange primer 2	GCCTACTGCAGCTGCATCTGGAATGAGTCC
ASF1b S169A Q5 primer 1	CCAGGACCCCGCCCTGGGCTGCG
ASF1b S169A Q5 primer 2	GTCTCTATGGCCTCCAGCCTGTCCATGTTG
ASF1b S198A Q5 primer 1	CCCTGAGAACGCCATGGACTGCATC
ASF1b S198A Q5 primer 2	AGGAGGCCAGGGATGCAG
ASF1b E196A Q5 primer 1	CCTCCTCCCTGCGAACTCCATGG
ASF1b E196A Q5 primer 2	CCAGGGATGCAGCCAGGG
ASF1b N197A Q5 primer 1	CCTCCCTGAGGCCTCCATGGACTGCATCTAAG
ASF1b N197A Q5 primer 2	AGGCCAGGGATGCAGCCA
ASF1b M199A Q5 primer 1	TGAGAACTCCGCGGACTGCATCTAAGCGG
ASF1b M199A Q5 primer 2	GGGAGGAGGCCAGGGATG
TLK2 sg4 primer1	CACCGCAGTAAATGAGGCTCCGTGT
TLK2 sg4 primer2	AAACACACGGAGCCTCATTTACTGC

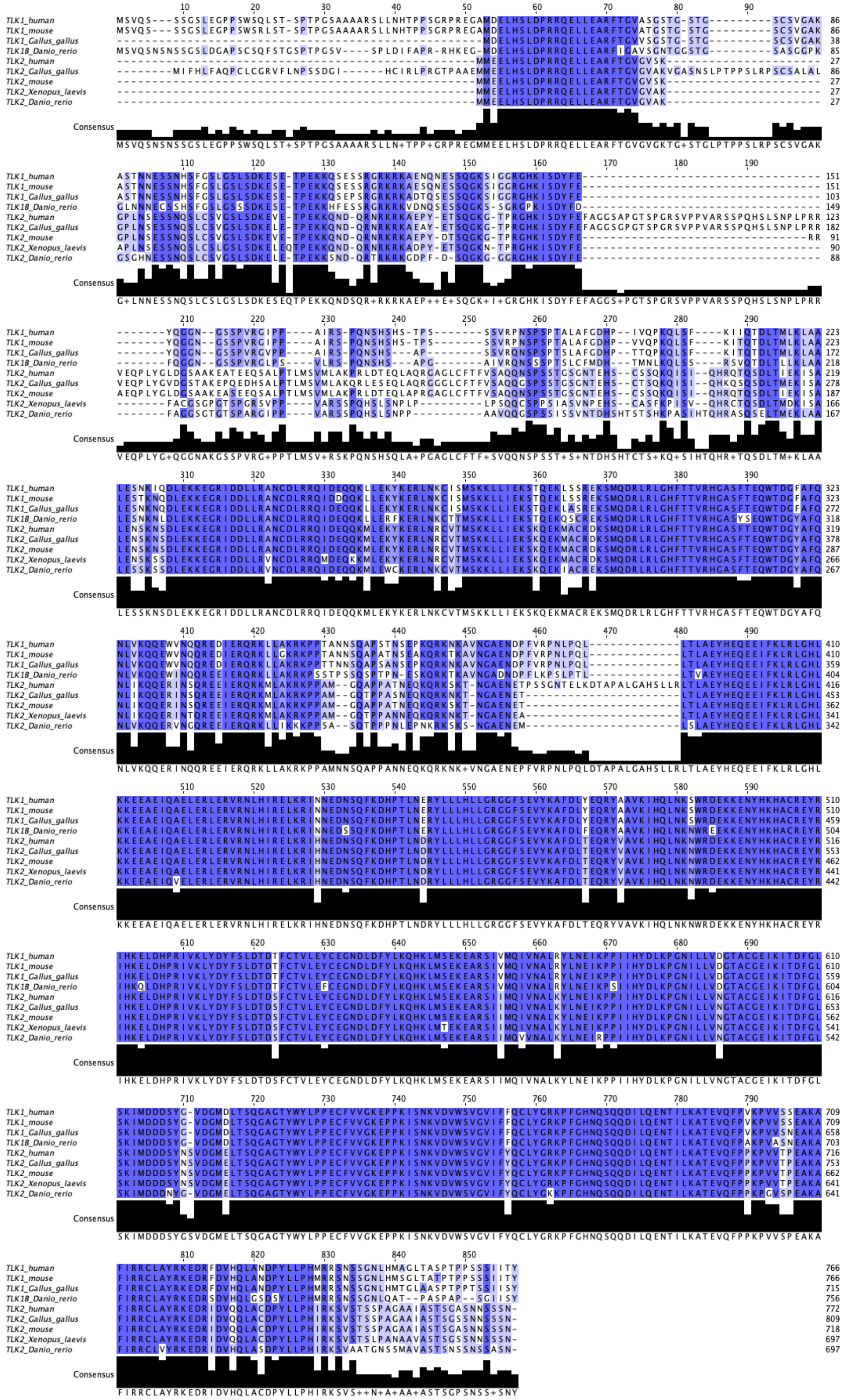

Supplementary Figure 1. Alignment of protein sequences of TLK1 and TLK2 from a range of animal species. Conserved residues are indicated by blue highlighting and in the histogram at the bottom of each row.

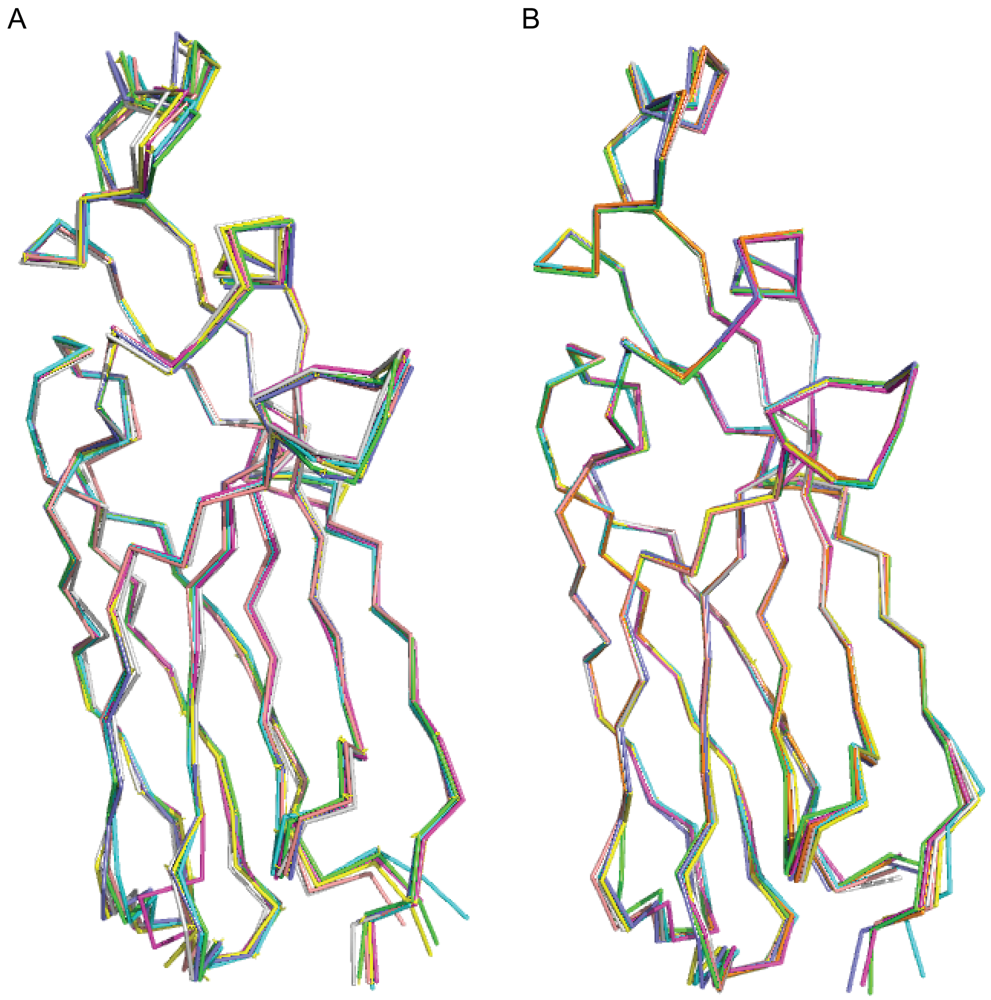

Supplementary Figure 2. The ASF1a structure exhibits little variation between the chains in each crystal structure. (**A**) The 7 chains in the apo-ASF1a crystal structure were superimposed and displayed in ribbon format. Each chain is depicted in a different color. (**B**) The 8 ASF1a chains in the ASF1a·TLK2 peptide complex crystal structure were superimposed and displayed in ribbon format. Each chain is depicted in a different color.

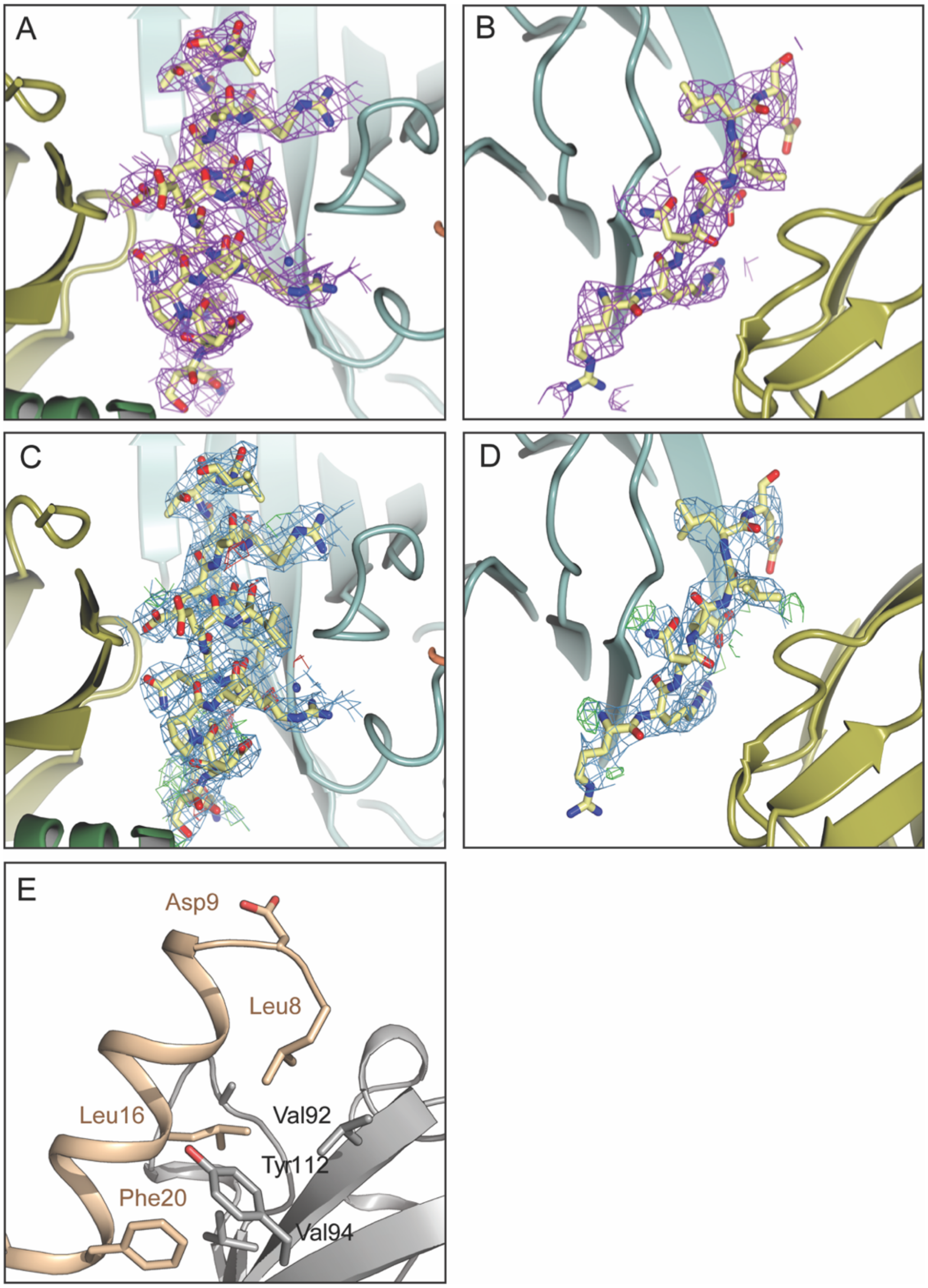

Supplementary Figure 3. Crystallographic analysis of the ASF1a-TLK2 complex. (**A,B**) Simulated annealing composite omit maps for TLK2 peptide binding ASF1a at the H3-binding site (**A**) and at the B-domain binding site (**B**) contoured at 1.06 sigma. ASF1 peptide shown in stick format and TLK2 shown in cartoon format in cyan. Crystallographic and symmetry related molecules of ASF1 shown in yellow and green. Image made using CCP4mg. (**C,D**) 2Fo-Fc and Fo-Fc electron density maps in same poses as A and B. 2Fo-Fc maps contoured at 1 sigma (blue) and Fo-Fc maps at +3 sigma (green) and -3 sigma (red). (**E**) Additional detail of TLK2 binding ASF1a at the H3-binding site in chains where Leu8 of TLK2 is visible.

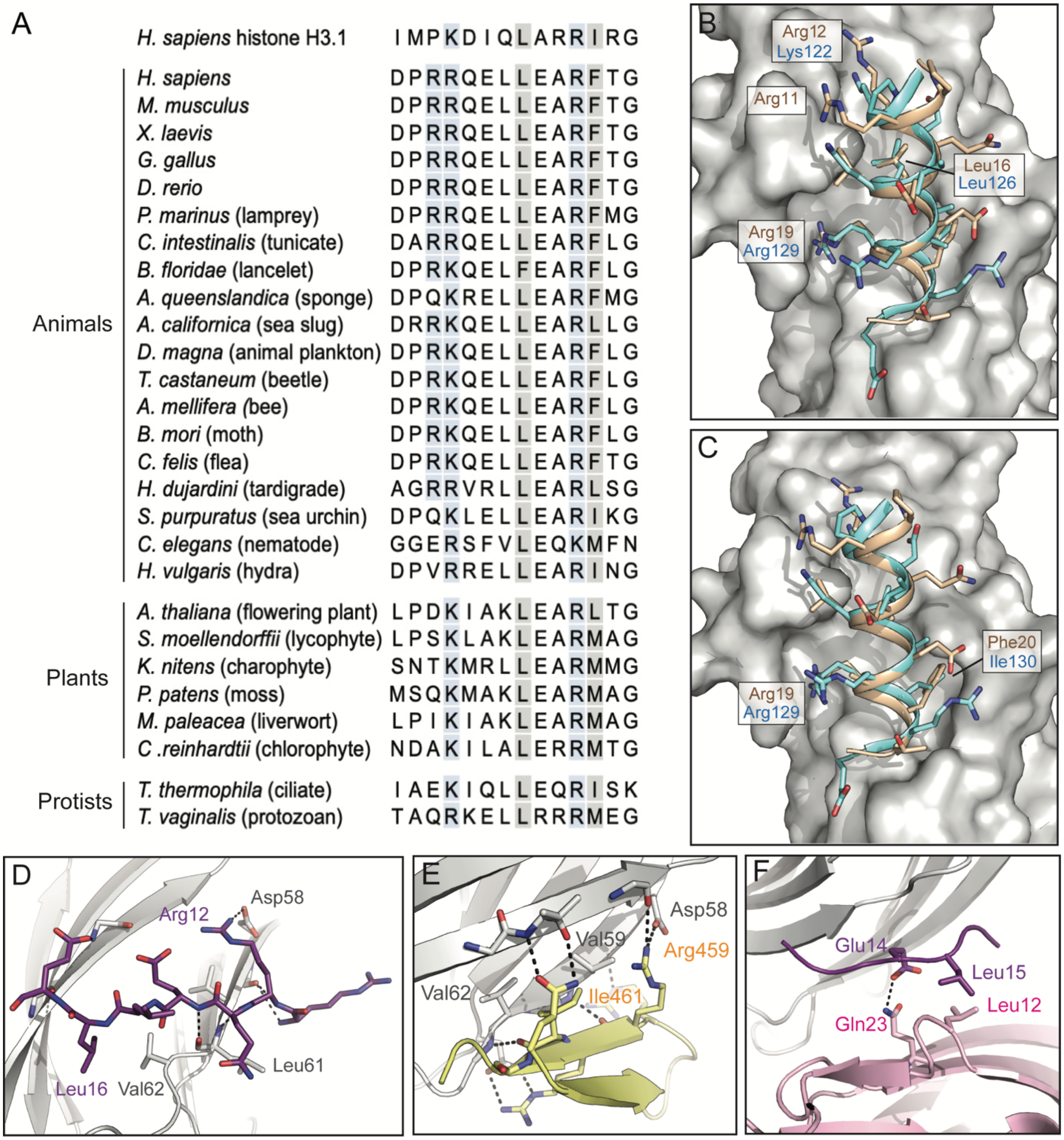

Supplementary Figure 4. Analysis of ASF1-TLK2 binding surfaces. (**A**) Alignment showing conservation of the TLK2 N-terminal ASF1-binding site and similarity to the corresponding region of human histone H3.1. Interface residues are shaded. Sequences are from either TLK2 orthologs or the sole TLK orthologs of the indicated organism. (**B,C**) The homologous region of histone H3 from PDB 2IO5 is shown overlaid on the ASF1a-TLK2 peptide structure. (**D,E**) Cartoon depicting details of the TLK2 peptide (purple) binding ASF1a (grey) at the HIRA-binding site (**D**) and comparison with HIRA (yellow) binding at the equivalent site (**E**). The ASF1a-HIRA complex is from PDB 2I32. (**F**) Depiction of TLK2 peptide (purple) binding between two ASF1a molecules (colored grey and pink) in the asymmetric unit.

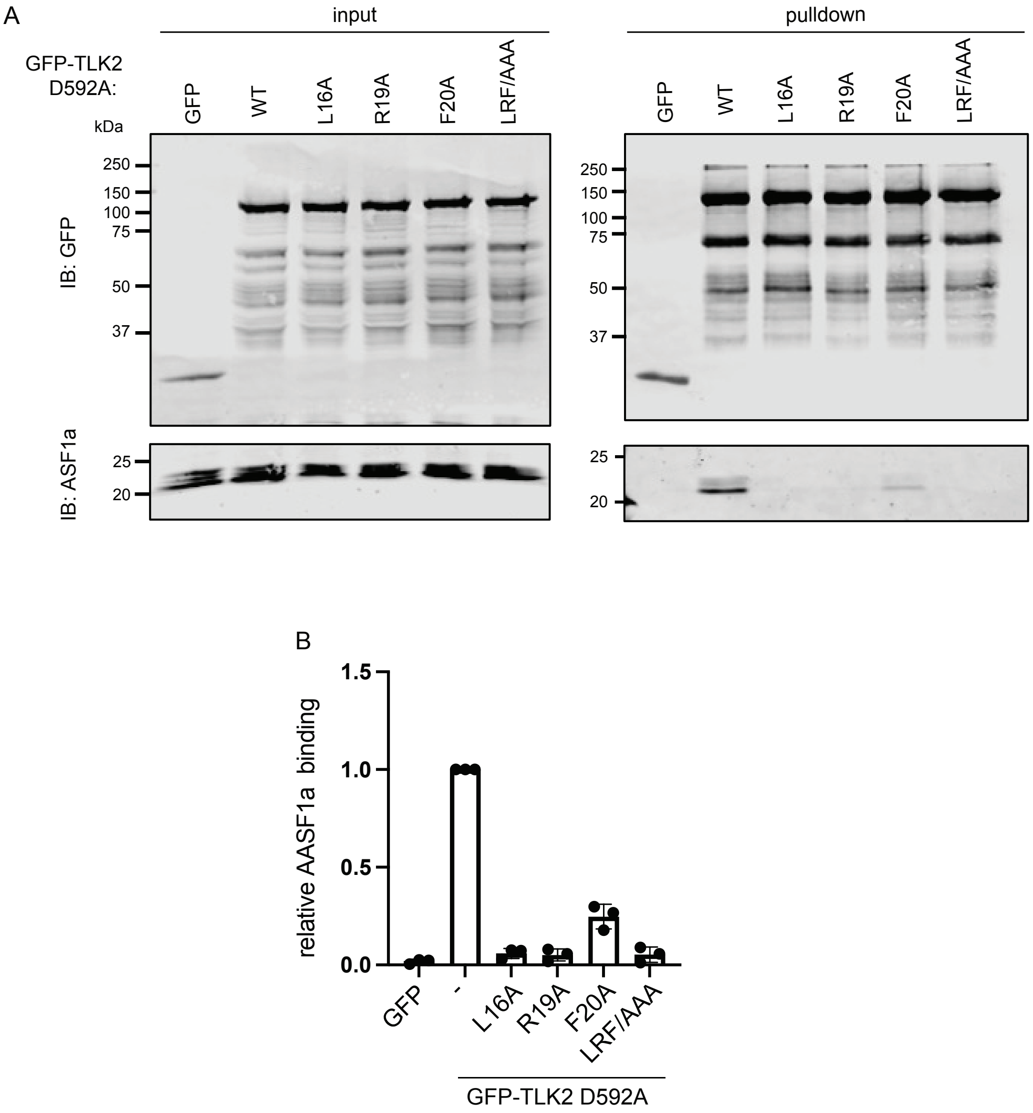

Supplementary Figure 5. Mutation of individual TLK2 residues strongly inhibit ASF1a binding. (A,B) HEK293T cells were transfected with GFP, GFP-tagged kinase-inactive TLK2 (D592A) or GFP-TLK2 (D592A) containing additional mutations at the structurally defined ASF1a-binding site and anti-GFP nanobody pulldown assays were performed. Precipitated GFP proteins and co-precipitating endogenous ASF1a were fractionated by SDS-PAGE and detected by immunoblotting. A representative experiment is shown in (A). Relative ASF1a binding normalized to GFP-TLK2 (D592A) was quantified in 3 independent replicates and mean ± S.D is shown in (B). Source data are provided as a Source Data file
