# Supplementary material for: Tousled-like kinase 2 targets ASF1 histone chaperones through client mimicry
Source: Nat Commun. 2022 Feb 8;13:749. doi: 10.1038/s41467-022-28427-0 (PMC8826447; doi:10.1038/s41467-022-28427-0)
Supplement: Supplementary file 3 — Reporting Summary [file 41467_2022_28427_MOESM3_ESM.pdf]

nature portfolio

Corresponding author(s): Benjamin E. Turk and David A. Calderwood

Last updated by author(s): Dec 27, 2021

Reporting Summary

Nature Portfolio wishes to improve the reproducibility of the work that we publish. This form provides structure for consistency and transparency in reporting. For further information on Nature Portfolio policies, see our Editorial Policies and the Editorial Policy Checklist.

Statistics

For all statistical analyses, confirm that the following items are present in the figure legend, table legend, main text, or Methods section.

n/a	Confirmed	
☐	☒	The exact sample size (*n*) for each experimental group/condition, given as a discrete number and unit of measurement
☐	☒	A statement on whether measurements were taken from distinct samples or whether the same sample was measured repeatedly
☐	☒	The statistical test(s) used AND whether they are one- or two-sided<br>*Only common tests should be described solely by name; describe more complex techniques in the Methods section.*
☐	☒	A description of all covariates tested
☐	☒	A description of any assumptions or corrections, such as tests of normality and adjustment for multiple comparisons
☐	☒	A full description of the statistical parameters including central tendency (e.g. means) or other basic estimates (e.g. regression coefficient) AND variation (e.g. standard deviation) or associated estimates of uncertainty (e.g. confidence intervals)
☐	☒	For null hypothesis testing, the test statistic (e.g. *F*, *t*, *r*) with confidence intervals, effect sizes, degrees of freedom and *P* value noted<br>*Give P values as exact values whenever suitable.*
☒	☐	For Bayesian analysis, information on the choice of priors and Markov chain Monte Carlo settings
☒	☐	For hierarchical and complex designs, identification of the appropriate level for tests and full reporting of outcomes
☒	☐	Estimates of effect sizes (e.g. Cohen's *d*, Pearson's *r*), indicating how they were calculated

Our web collection on statistics for biologists contains articles on many of the points above.

Software and code

Policy information about availability of computer code

Data collection	Gels and clonogenic assays were imaged on LiCor Odyssey scanner using Image Studio (Li-Cor version 5.2). Radioactive gels were imaged on a Bio-Rad FX imager using QuantityOne software.
Data analysis	Immunoblotting data were analyzed using LiCor Image Studio Lite v5.2 or with ImageJ , and phosphorimager scans were quantified using ImageJ.<br>Data were analyzed and statistical analysis performed with GraphPad Prism versions 8.2.1 and 9.3.0 as indicated in the manuscript or with Microsoft Excel Version 16.54<br>X-ray data was integrated using XDS (VERSION Mar 15, 2019 BUILT=20190606) and Aimless (v0.7.4). Structures determined and refined using Phenix suite (v1.19), Aimless (v0.7.4) Phaser (v2.8.2), PDB Extract (v3.27), Coot (v0.8.8), MOLPROBITY (v4.02b-467).<br>Multiple sequence alignments were performed using T-coffee, which can be downloaded from: https://www.tcoffee.org/Projects/tcoffee/index.html#DOCUMENTATION<br><br>Structure figures were generated using Pymol (Schrodinger, Version 2.4).

For manuscripts utilizing custom algorithms or software that are central to the research but not yet described in published literature, software must be made available to editors and reviewers. We strongly encourage code deposition in a community repository (e.g. GitHub). See the Nature Portfolio guidelines for submitting code & software for further information.

Data

Policy information about availability of data

All manuscripts must include a data availability statement. This statement should provide the following information, where applicable:

- Accession codes, unique identifiers, or web links for publicly available datasets
- A description of any restrictions on data availability
- For clinical datasets or third party data, please ensure that the statement adheres to our policy

Data availability
Structural coordinates were deposited in the Protein Data Bank (www.wwpdb.org) under the accession codes PDB: 7LNY (Apo-ASF1a) and PDB: 7LO0 (ASF1a/TLK2 peptide complex). Constructs and reagents are available from the authors upon request. Source data are provided with this paper.

Field-specific reporting

Please select the one below that is the best fit for your research. If you are not sure, read the appropriate sections before making your selection.

[x] Life sciences [ ] Behavioural & social sciences [ ] Ecological, evolutionary & environmental sciences

For a reference copy of the document with all sections, see nature.com/documents/nr-reporting-summary-flat.pdf

Life sciences study design

All studies must disclose on these points even when the disclosure is negative.

Sample size	At least 3 independent replicates were performed because that is the standard accepted requirement in the field. When more experiments were performed all data were included in figures
Data exclusions	data were not excluded
Replication	All data could be replicated. The number of independent replicates of each experiment is reported in figure legends. The PSPL data was repeated twice but all other experiments were performed at least 3 times. All attempts at replication were successful.
Randomization	Randomization is not appropriate for the experiments performed here
Blinding	Experimenters were not blinded as this was not appropriate for these biochemical assays - results are quantitatively assessed after scanning gels

Reporting for specific materials, systems and methods

We require information from authors about some types of materials, experimental systems and methods used in many studies. Here, indicate whether each material, system or method listed is relevant to your study. If you are not sure if a list item applies to your research, read the appropriate section before selecting a response.

Materials & experimental systems

n/a	Involved in the study	
[ ]	[x]	Antibodies
[ ]	[x]	Eukaryotic cell lines
[x]	[ ]	Palaeontology and archaeology
[x]	[ ]	Animals and other organisms
[x]	[ ]	Human research participants
[x]	[ ]	Clinical data
[x]	[ ]	Dual use research of concern

Methods

n/a	Involved in the study	
[x]	[ ]	ChIP-seq
[x]	[ ]	Flow cytometry
[x]	[ ]	MRI-based neuroimaging

Antibodies

Antibodies used	anti-GFP (Rockland, catalogue #600-101-215), anti-Flag (Sigma-Aldrich, #F1804) anti-ASF1a (Cell Signaling Technology, C6E10 Rabbit mAb #2990) anti-TLK2 (Santa Cruz, sc-393506). Secondary antibodies conjugated to fluorescent IR Dye800 or IR Dye680 were purchased from LI-COR Biosciences and used at 1:25000 dilution in 5% non-fat milk in TBS-T (IRDye 680RD goat anti-mouse IgG, catalogue #92668070; IRDye 800CW donkey anti-mouse IgG, catalogue #92632212; IRDye 680RD donkey anti-rabbit IgG, catalogue #92632213; IRDye 800CW donkey anti-rabbit IgG, catalogue #926680723; IRDye 680RD donkey anti-goat IgG, catalogue #92668074)
Validation	Antibodies were obtained commercially and validated as detecting proteins of the predicted sizes. Additional controls include the

acquisition of signal at the expected size in transfected samples (anti-FLAG, anti-GFP, ASF1 and TLK2) or loss of signal following knockout (TLK2) (e.g., Fig 7 of manuscript)

Eukaryotic cell lines

Policy information about cell lines

Cell line source(s)	MCF7 - original source ATCC<br>HEK-293T - original source ATCC
Authentication	Cell lines were not re-authenticated since purchase. Conclusions in the manuscript do not rest on the cell line identity.
Mycoplasma contamination	All cell lines were routinely tested negative for mycoplasma using MycoAlert (Lonza Bio)
Commonly misidentified lines<br>(See ICLAC register)	We did not use commonly misidentified cell lines but, in any case, conclusions in the manuscript are not contingent upon the specific identity of cell line used.
